# Evaluating of radiation-induced cerebral contrast enhancements in brain metastases: analysis of incidence and risk predictors

**DOI:** 10.1007/s10585-025-10372-z

**Published:** 2025-09-19

**Authors:** Kerem Tuna Tas, Abduljalil Sheirieh, Phillip Lishewski, Fatima Frosan Sheikhzadeh, Edgar Smalc, Ioanna Fragkandrea-Nixon, Khaled Elsayad, Klemens Zink, Hilke Vorwerk, Sebastian Adeberg, Ahmed Gawish

**Affiliations:** 1 Department of Radiotherapy and Radiation Oncology, Marburg University Hospital, 35043 Marburg, Germany; 2 Department of Radiotherapy and Radiation Oncology, Philipps-Universität Marburg, Marburg, Germany; 3 Marburg Ion-Beam Therapy Center (MIT), Department of Radiotherapy and Radiation Oncology, Marburg University Hospital, Marburg, Germany; 4 University Cancer Center (UCT) Frankfurt – Marburg, Marburg, Germany; 5 LOEWE Research Cluster for Advanced Medical Physics in Imaging and Therapy, (ADMIT), TH Mittel Hessen University of Applied Sciences, Giessen, Germany; 6 The Beatson West of Scotland Cancer Center, Glasgow, UK

**Keywords:** Stereotactic radiation therapy, Radiosurgery, Radiation oncology

## Abstract

Radiation-induced cerebral contrast enhancements (RICE) are frequent after photon and particularly proton radiation therapy (RT) and are associated with a significant risk for neurologic morbidity. While stereotactic radiosurgery (SRS) and fractionated stereotactic radiotherapy (FSRT) provide high rates of local control (LC), RICE remains a significant concern that may impact patient outcomes and quality of life. This study aims to assess the incidence, risk factors, and clinical implications of RICE in patients treated with SRS or FSRT for brain metastases (BMs). A retrospective analysis was conducted on 175 patients with 330 BMs treated between October 2015 and November 2023. Median follow-up (FU) was 17 months. The incidence of RICE was determined, and potential predictive factors were evaluated using univariate Cox regression analysis. RICE was identified in 8 patients with 10 lesions (3%). Systemic therapy without immunotherapy (IT) was found to be a significant predictor of RICE (HR = 4.161, * p*= 0.027). No significant association was observed between the occurrence of RICE and overall survival (OS), indicating that while RICE is a treatment-related adverse event, it does not appear to significantly influence long-term survival. RICE occurs in a small subset of patients treated with SRS or FSRT. Systemic therapy without IT significantly increases the risk, underscoring the need for careful treatment planning and patient selection. While necrosis does not impact overall survival, its potential effects on neurological function and quality of life warrant continued research into preventive and management strategies.

## Introduction

Stereotactic radiosurgery (SRS) and fractionated stereotactic radiotherapy (FSRT) have become cornerstone treatments for brain metastases (BMs), offering high rates of local control (LC) with minimal damage to surrounding healthy tissue [1, 2] These techniques deliver highly focused radiation to tumor targets while sparing adjacent brain structures, making them particularly effective for patients with limited metastatic disease [3]. However, despite their precision, radiation-induced cerebral contrast enhancement (RICE) remains a significant concern, with reported incidences ranging from 3 to 24% depending on treatment parameters and patient characteristics [4, 5].

RICE is a delayed complication that typically occurs months to years after treatment and is characterized by inflammation, vascular injury, and demyelination in the irradiated brain tissue [6]. The clinical presentation of RICE can vary widely, from asymptomatic imaging findings to severe neurological deficits, including cognitive decline, motor dysfunction, and seizures [7]. These symptoms can significantly impact a patient's quality of life, making the prevention and management of RICE a critical area of research in neuro-oncology.

The pathophysiology of RICE is complex and multifactorial, involving direct damage to endothelial cells, disruption of the blood–brain barrier, and activation of inflammatory pathways [8]. High radiation doses, large treatment volumes, and re-irradiation are known risk factors for RICE, but patient-specific factors such as systemic therapies and underlying comorbidities may also play a role [9]. Advances in imaging and treatment planning, including the use of magnetic resonance imaging (MRI) and customized stereotactic masks, have reduced the incidence of RICE, but it remains a challenging complication to predict and manage [10].

This study aims to evaluate the incidence and predictors of RICE in a cohort of patients treated with SRS or FSRT for BMs. By analyzing treatment parameters, patient characteristics, and clinical outcomes, we seek to provide insights into the factors contributing to RICE and its impact on patient survival. Understanding these factors is essential for optimizing treatment strategies, improving patient outcomes, and minimizing the risk of this debilitating complication.

## Materials and methods

### Patient selection

This retrospective study was conducted following approval from the institutional ethics committee. A review of medical records identified patients with BMs who underwent SRS and FSRT at our Department of Radiation Oncology between October 2015 and November 2023. A total of 175 patients with 330 BMs met the inclusion criteria. To ensure reliable follow-up (FU) data, only patients with a minimum FU period of three months were included. Patients with two distinct primary carcinomas were excluded.

The analysis included both primary stereotactic radiotherapy (SRT) cases and those treated postoperatively with adjuvant SRT. The selection of patients for either primary or adjuvant SRT was based on multidisciplinary evaluation by the neuro-oncology tumor board. Primary SRT was recommended for cases with deep-seated, inoperable, small, or asymptomatic metastases, as well as those located in critical functional brain areas. Surgical resection was prioritized in cases of symptomatic, accessible metastases or when histological confirmation was necessary.

### Radiotherapy planning and treatment

Treatment planning was conducted using computed tomography (CT) imaging, supplemented with contrast-enhanced MRI at a 1–2 mm slice thickness. All patients were immobilized with customized stereotactic masks to maintain precise isocenter positioning throughout the treatment process. RT planning was performed using the STP 3D-planning system (Stryker-Leibinger, Freiburg, Germany). Each tumor target was manually delineated to ensure accurate treatment delivery.

Radiation therapy was administered using a linear accelerator (Siemens, Erlangen, Germany). Before each session, isocenter verification was performed to maintain treatment accuracy. The prescribed radiation dose was delivered to the isocenter, covering the entire tumor volume. For primary SRT, the gross tumor volume (GTV) was determined based on MRI scans, whereas for patients who had undergone prior surgery, the clinical target volume (CTV) included the entire resection cavity and any residual contrast-enhancing areas. The planning target volume (PTV) was defined by expanding the target volume by 1–2 mm for SRS and 3–5 mm for FSRT.

Dose adjustments were made based on tumor characteristics and the proximity to critical brain structures. In most cases, doses were prescribed to the 80% isodose line, with 95% isodose prescriptions applied for larger or irregularly shaped lesions. FSRT was preferred over SRS for lesions larger than 2 cm or those in direct contact with the ventricular system. Beam delivery was optimized using a micro-multileaf collimator (2 mm leaf width at the isocenter). To reduce the risk of radiation-induced cerebral edema, intravenous corticosteroids were administered one hour before treatment and four hours after treatment.

For patients who developed post-treatment contrast enhancements suspected to represent RICE, detailed dosimetric review was performed. Retrospective analysis of radiation plans was conducted to confirm lesion localization within high-dose areas (≥ 80% isodose), and parameters such as biologically effective dose (BED), PTV and number of treatment fractions were correlated with the clinical and radiological diagnosis of RICE [11].

### Post-treatment follow-up

Patients underwent their first clinical evaluation six weeks after RT, with MRI scans scheduled at three months post-treatment. FU imaging was conducted every three to six months, depending on individual clinical needs. In cases of suspected tumor recurrence, patients were re-evaluated by the neuro-oncology tumor board to assess potential salvage treatment options. Adverse effects were categorized based on the Common Terminology Criteria for Adverse Events (CTCAE). MRI imaging was further used to detect post-treatment radiation effects, including edema and suspected RICE.

### Diagnosis and classification of radiation-induced cerebral contrast enhancements

Suspected cases were systematically documented, with additional diagnostic and treatment-related data retrospectively verified.The diagnosis was established based on clinical presentation and imaging criteria, with histopathological confirmation when surgical resection was performed. To distinguish RICE from tumor progression, a five-tier classification system was employed:

- Standard MRI Evaluation: Diagnosis was based on contrast-enhanced MRI and fluid-attenuated inversion recovery (FLAIR) sequences, identifying enlarging lesions that exhibited regression with steroid therapy without the need for additional interventions.
- MR Perfusion Imaging: Perfusion-weighted MRI was used in combination with conventional MRI findings to enhance diagnostic accuracy.
- Histopathological Confirmation: Surgical biopsy was performed in select cases, confirming predominantly necrotic tissue with minimal or absent viable tumor cells.

In addition, all suspected RICE cases were evaluated based on the RANO (Response Assessment in Neuro-Oncology) criteria [11] Lesions were verified to lie within the 80% isodose volume according to the original treatment plan [12], and diagnoses were confirmed during multidisciplinary neuro-oncology board meetings, including specialists in radiation oncology, neuroradiology, and neurosurgery.

To support the diagnosis of RICE and demonstrate its spatial relationship to the irradiated volume, representative MRI images before and after radiotherapy were retrospectively selected. These images include T1-weighted contrast-enhanced sequences with the original isodose lines from treatment planning. This visual correlation between dose distribution and lesion location was used to confirm the presence of RICE within high-dose areas (≥ 80% isodose) (Figs. 6 and 7).

### Statistical analysis

Statistical analysis was conducted using SPSS version 29 (IBM, Armonk, NY, USA), with a significance threshold set at *p* < 0.05. Pairwise deletion was applied to address missing data points. The dataset was structured in long format to account for cases where patients underwent multiple SRT sessions.

Patient survival following radiosurgery was estimated using the Kaplan–Meier method, with censoring at the final FU date (data collection ended on January 1, 2025). The incidence of RICE was determined using the cumulative incidence competing risk method, analyzed on a per-lesion basis. Competing risks included patient mortality, salvage surgery, or additional radiosurgery for recurrent disease. In cases where patients underwent repeat radiosurgery, the time to necrosis was measured from the date of the second radiosurgery session.

### Predictors of radiation-induced cerebral contrast enhancements

To assess the factors influencing RICE risk, several treatment parameters were evaluated, including the number of radiation beams (shots) and prescription isodose levels, which were found to be colinear with other key radiosurgery metrics such as radiation dose, conformity index, and target volume. For patients who received repeat radiosurgery, treatment variables from the second radiosurgical procedure were used in the analysis. Additionally, the time to retreatment was included as a continuous variable in the statistical models.

The relationship between clinical parameters and treatment outcomes was analyzed using Cox proportional hazards regression models, evaluating predictors of in-field progression (IFP) and out-field progression (OFP). Univariate analysis was initially performed, followed by multivariate regression models, incorporating clinically relevant or statistically significant variables. Given the limited number of IFP events, a forward likelihood ratio selection method was used to identify predictors of IFP. Conversely, due to the higher frequency of OFP events, a backward likelihood ratio selection method was applied for OFP analysis.

To compare outcomes between patients with and without prior surgery, chi-square tests were used to assess differences in primary tumor type and whether single vs. multiple metastases were present at the time of SRT.

## Results

### Patient characteristics

The clinical and demographic details of the study cohort are summarized in Table 1. A total of 175 patients with 330 BMs were included, comprising 95 females (54.3%) and 80 males (45.7%). The median age at first radiotherapy (RT) was 61 years and the mean FU duration was 25.8 months**.**

**Table 1:** Characteristics of the Cohort (n = 175)

Characteristic	Value	Details
Age at first radiotherapy	61 years	Range: 22–87 years, SD: ± 10.6
Sex	Male	80 (45.7%)
	Female	95 (54.3%)
Primary tumor type	Non-small cell lung cancer	67 (38.3%)
	Melanoma	39 (22.3%)
	Breast cancer	27 (15.4%)
	Renal cell carcinoma	14 (8.0%)
	Colorectal cancer	18 (10.3%)
	Esophageal cancer	4 (2.3%)
	Urothelial cancer	4 (2.3%)
	Ovarian cancer	2 (1.1%)

*SD* = standard deviation

±  = respective means ± SD

The most common primary tumor types were non-small cell lung cancer (NSCLC) (38.3%), malignant melanoma (MM) (22.3%), breast cancer (BC) (15.4%), colorectal cancer (CRC) (10.3%), and renal cancer (RC) (8%)**.**

Most metastases were located in supratentorial regions (261 lesions; 79.1%), followed by the cerebellum (60 lesions; 18.2%) and brainstem (9 lesions; 2.7%). At the time of treatment, 178 metastases (53.9%) were solitary, while 152 (46.1%) were multiple, with a mean of three metastases treated per course**.**

Regarding previous treatments, 47 patients (14.2%) had received prior RT for BMs, including SRT in 5.1% and whole-brain radiotherapy (WBRT) in 9.1%. Surgical resection had been performed in 87 cases (26.4%), while 243 (73.6%) had no prior surgery**.**

Concerning radiotherapy modality, 206 metastases (62.4%) received single-fraction stereotactic radiosurgery (SRS), with a median prescribed dose of 20 Gy to the 80% isodose line. For larger lesions or re-irradiation, dose modifications included 18 Gy and, in five cases, 10–15 Gy**.**

The remaining 124 metastases (37.6%) were treated with FSRT. The median FSRT dose was 30 Gy, typically delivered in 4–25 fractions**,** with 62.3% of FSRT cases receiving 30 Gy in 10 fractions, reflecting institutional practice until 2021**.** Of the FSRT group, 87 metastases (70.2%) received adjuvant therapy following total or subtotal resection.

Concomitant systemic therapy was administered in 52.1% of cases, and 34.8% of patients received immunotherapy (IT)**.** A detailed overview of metastasis characteristics, treatment approaches, and outcomes is provided in Tables 2 and 3.

**Table 2:** Characteristics and treatment of the 330 Metastases

Characteristic	Value	Details
Previous radiotherapy	None	283 (85.8%)
	Stereotactic radiotherapy	17 (5.1%)
	Whole-brain radiotherapy	30 (9.1%)
Previous neurosurgical resection	None	243 (73.6%)
	Tumor resection	87 (26.4%)
Singular metastasis	Yes	178 (53.9%)
Multiple metastases	Yes	152 (46.1%)
Multiple metastases (mean)	3	Range: 2–6
Radiotherapy modality	Stereotactic radiosurgery	206 (62.4%)
	Fractionated stereotactic radiotherapy	124 (37.6%)
Stereotactic radiosurgery dose	Median: 20 Gy	Range: 10–20 Gy, SD: ± 1.63 Gy
Fractionated stereotactic radiotherapy dose	Median: 30 Gy	Range: 21–54 Gy, SD: ± 5.2 Gy
Fractionated stereotactic radiotherapy fractions		Range: 4–25 fractions
Systemic therapy at time of Stereotactic radiosurgery	Yes	46 (34.8%)
Systemic therapy overall	None	172 (52.1%)
	Yes	158 (47.9%)
Radiotherapy under immunotherapy	No	215 (65.2%)
	Yes	115 (34.8%)
Location	Supratentorial	261 (79.1%)
	Cerebellum	60 (18.2%)
	Brain stem	9 (2.7%)

*SD* = standard deviation

±  = respective means ± SD

**Table 3:** Treatment Outcomes of the 330 Metastases

Characteristic	Value
Follow-up duration, mean (range, SD)	25.8 months (3–208, ± 24.5)
In-field progression, number (%)	22 (6.7%)
Time to in-field progression, mean (range, SD)	10.3 months (2–26, ± 7.2)
Out-of-field progression, number (%)	95 (43.7%)
Time to out-of-field progression, mean (range, SD)	13.2 months (2–74, ± 13.8)
Radiation necrosis, number (%)	10 (3%)
Salvage therapy, number (%)	
Whole-brain radiotherapy	46 (13.9%)
Stereotactic radiotherapy	71 (21.6%)
Surgery	6 (1.8%)
Systemic therapy	8 (2.4%)

SD = standard deviation

±  = respective means ± SD

Among the 8 patients (10 lesions) who developed RICE, systemic therapy regimens prior to radiotherapy were as follows: two patients received no systemic therapy, one patient with two lesions received trastuzumab, one patient received crizotinib and alectinib, one patient with two lesions received osimertinib, one patient received nivolumab and ipilimumab, one patient received pembrolizumab, and one patient received ipilimumab.

During radiotherapy, one patient with two lesions continued trastuzumab, one patient received pembrolizumab, and one patient received ipilimumab. The patients who had been receiving crizotinib and alectinib, osimertinib, or nivolumab and ipilimumab before radiotherapy did not continue these treatments during radiotherapy. Five patients were not receiving any systemic therapy during radiotherapy.

All lesions were treated with single-fraction SRS, prescribed as 20 Gy to the 80% isodose line. The consistency of radiation parameters across RICE cases supports the potential role of systemic therapy as an influencing factor.

### Treatment outcomes

Over a median FU of 17 months**,** IFP was observed in 22 metastases (6.7%)**,** with a mean time to progression of 10.3 months **(**range: 2–26 months; SD ± 7.2), as reported in Table 3. OFP occurred in 95 metastases (43.7%)**,** with a mean time to progression of 13.2 months (range: 2–74 months; SD ± 13.8).

Neurocranial progression (NCP)—defined as any progression within the central nervous system (either IFP or OFP)—was observed in 163 patients (49.4%), with a mean time to progression of 11 months (range: 2–208 months; SD ± 22.7).

**Fig. 1:**
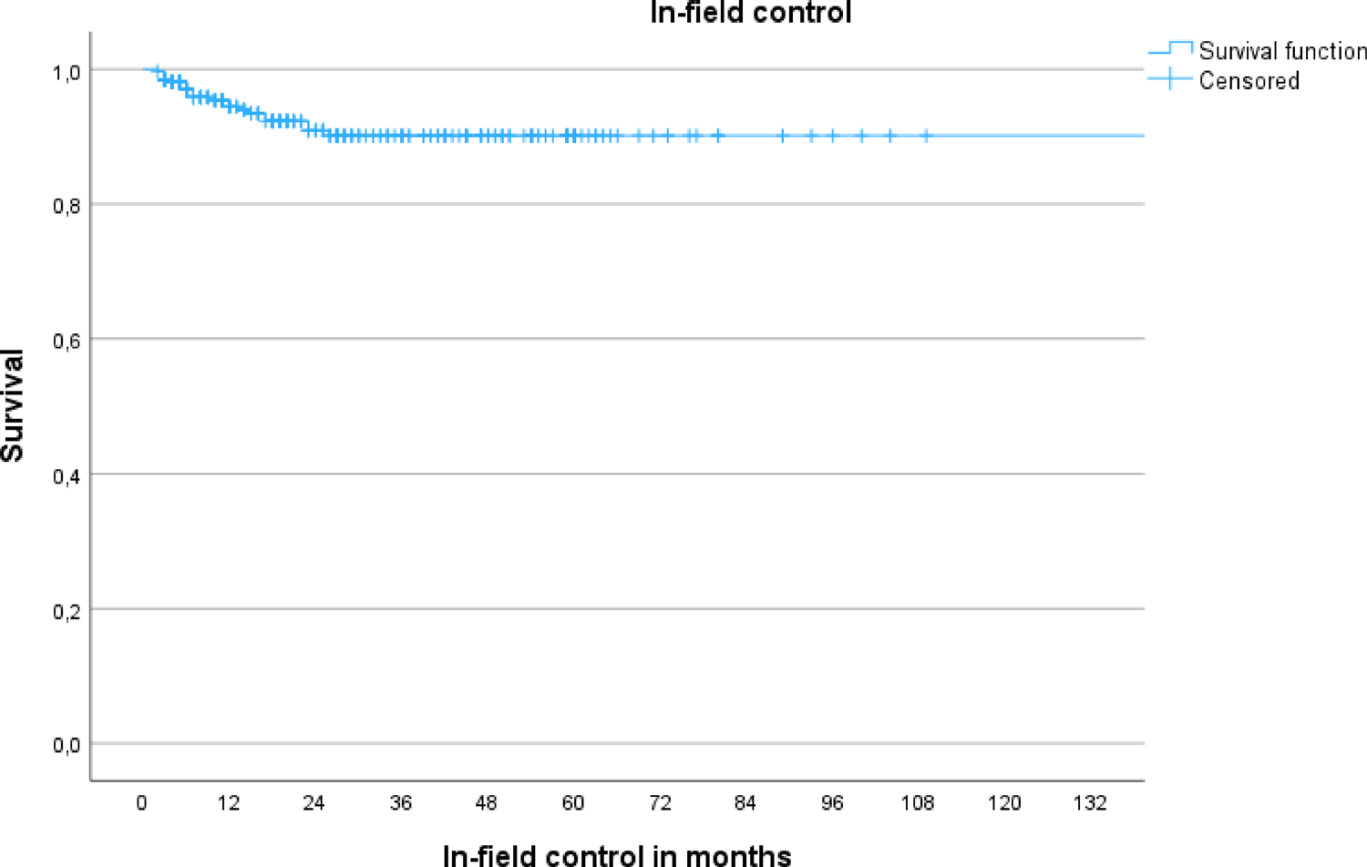
This diagram illustrates local failure after radiosurgery for the study cohort

**Fig. 2:**
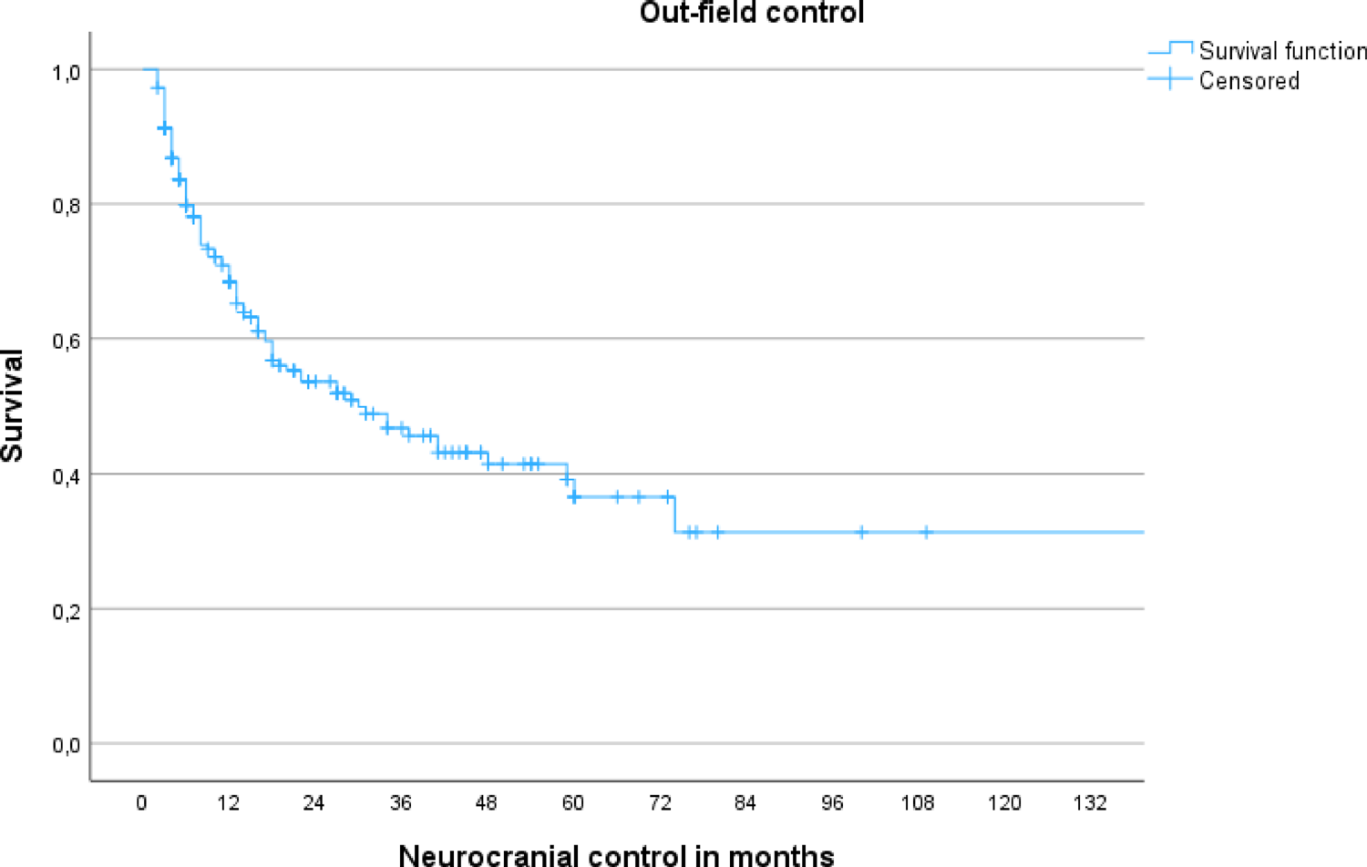
Kaplan Meier illustrates cranial progress after radiosurgery for the study cohort. Kaplan Meier demonstrates the Actuarial incidence of RICE, stratified by location

Figures 1 and 2 illustrate Kaplan–Meier survival curves for IFP and OFP/NCP, respectively, based on the 330 treated metastases.

In cases of progression, salvage therapy was applied based on clinical judgment. WBRT was administered in 46 cases (13.9%), while SRT was used in 71 cases (21.6%). Surgical resection was performed in 6 patients (1.8%), and systemic therapy was introduced in 8 cases (2.4%). The remaining patients received best supportive care (BSC). RICE was detected in 10 lesions (3%).

Overall, adverse events were rare and mild. Only one patient experienced Grade III fatigue, and no other Grade III or higher treatment-related toxicities were reported according to CTCAE criteria.

The mean overall survival (OS) for the entire cohort was 31.4 months (standard deviation ± 29.4), with a median OS of 21.5 months (range: 3–209 months). Cox regression analysis showed no statistically significant association (*p* = 0.37). The mean overall survival among patients who developed RICE was 26.1 months (standard deviation ± 23.4), with a median OS of 15.0 months (range: 6–66 months).

### Predictors of radiation-induced cerebral contrast enhancements (RICE)

Univariate Cox regression analysis was conducted to identify clinical and treatment-related variables associated with the development of RICE. Among all factors assessed, systemic therapy without IT was found to be the only statistically significant predictor of RICE**,** with a hazard ratio (HR) of 4.161 and a *p*-value of 0.027 (95% CI 1.172–14.780). This finding suggests that patients undergoing systemic treatment without the immunomodulatory benefits of IT are at a substantially higher risk of developing RICE (Table 4).

**Table 4:** Univariate Cox regression analysis of predictors of RICE

Predictor	HR	p-value	95% CI
Adjuvant RT	0.03	0.241	0.00–10.608
Previous irradiation	0.699	0.734	0.88–5.520
Radiotherapy under immunotherapy	0.733	0.631	0.207–2.599
Systemic therapy	4.571	0.055	0.97–21.534
Planning Target Volume	0.914	0.178	0.801–1.042
Sex	0.368	0.415	0.033–4.072
Age at radiotherapy	0.986	0.621	0.931–1.044
Biological Effective Dose	1.237	0.096	0.963–1.589
Brain metastases ≥ 4	0.039	0.653	0–52623.319
Systemic therapy without immunotherapy	4.161	0.027	1.172–14.780
Whole-Brain Radiotherapy	1.612	0.651	0.203–12.772
Number of metastases	1.093	0.586	0.794–1.503
Radiotherapy modality (SRS/FSRT)	0.163	0.163	0–4.633
Single or multiple RT courses	3.408	0.076	0.878–13.229
Primary tumor type	0.763	0.162	0.522–1.115
Localization (Supratentorial or Cerebellum)	1.023	0.64	0.931–1.124

**Fig. 3:**
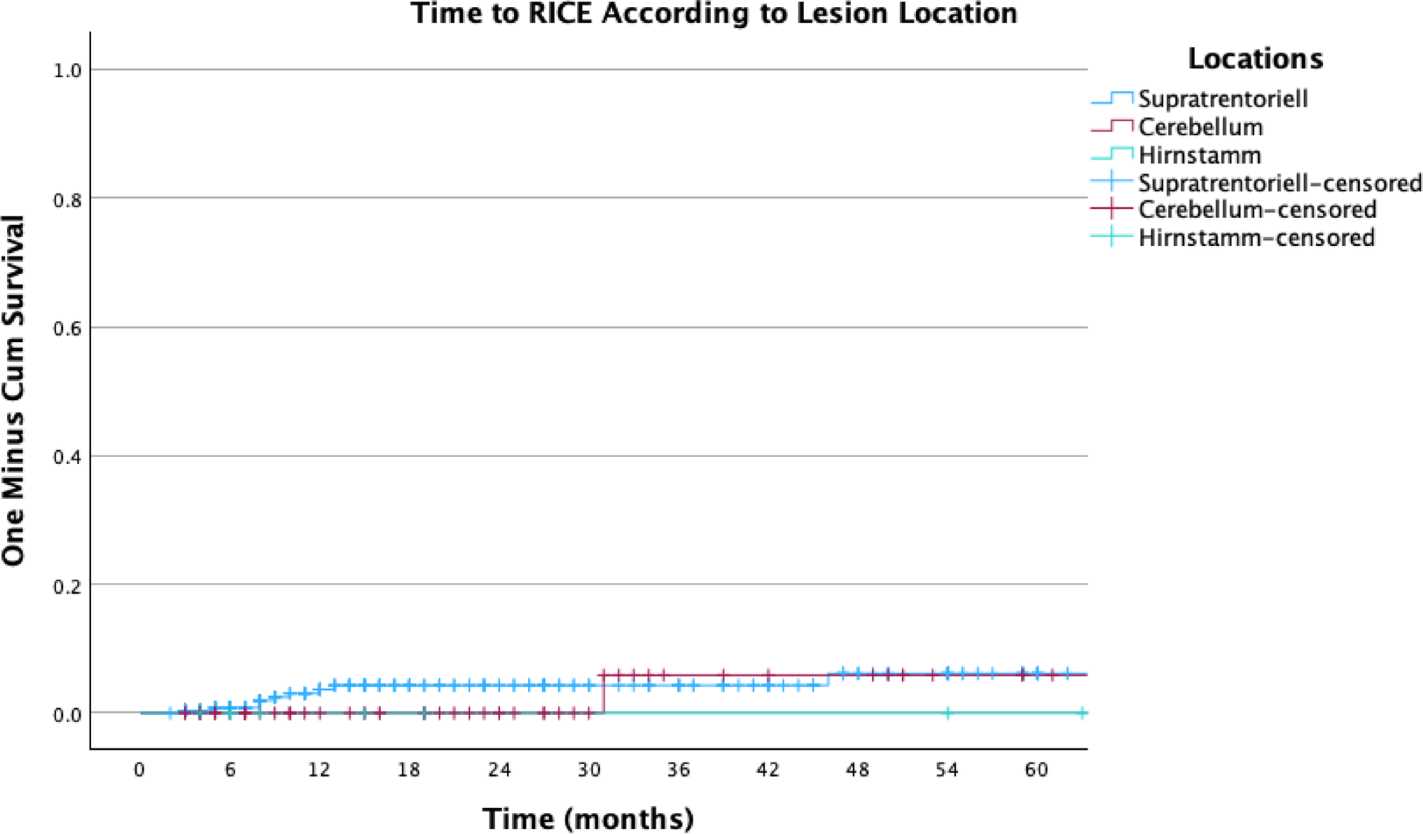
Actuarial incidence of RICE by brain locations

**Fig. 4:**
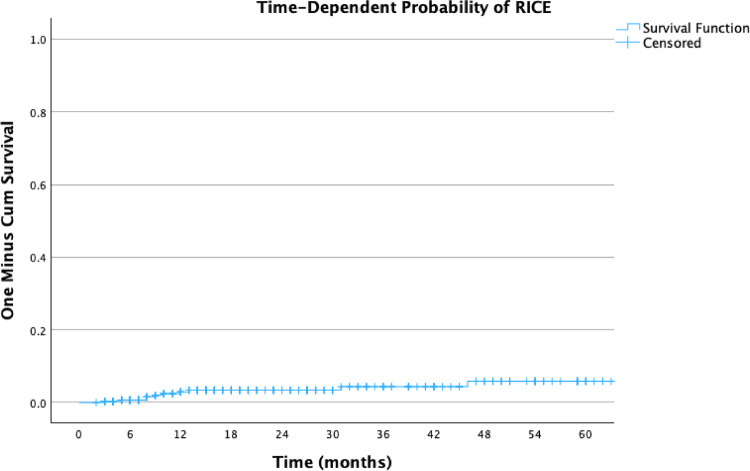
Actuarial incidence of RICE

**Fig. 5:**
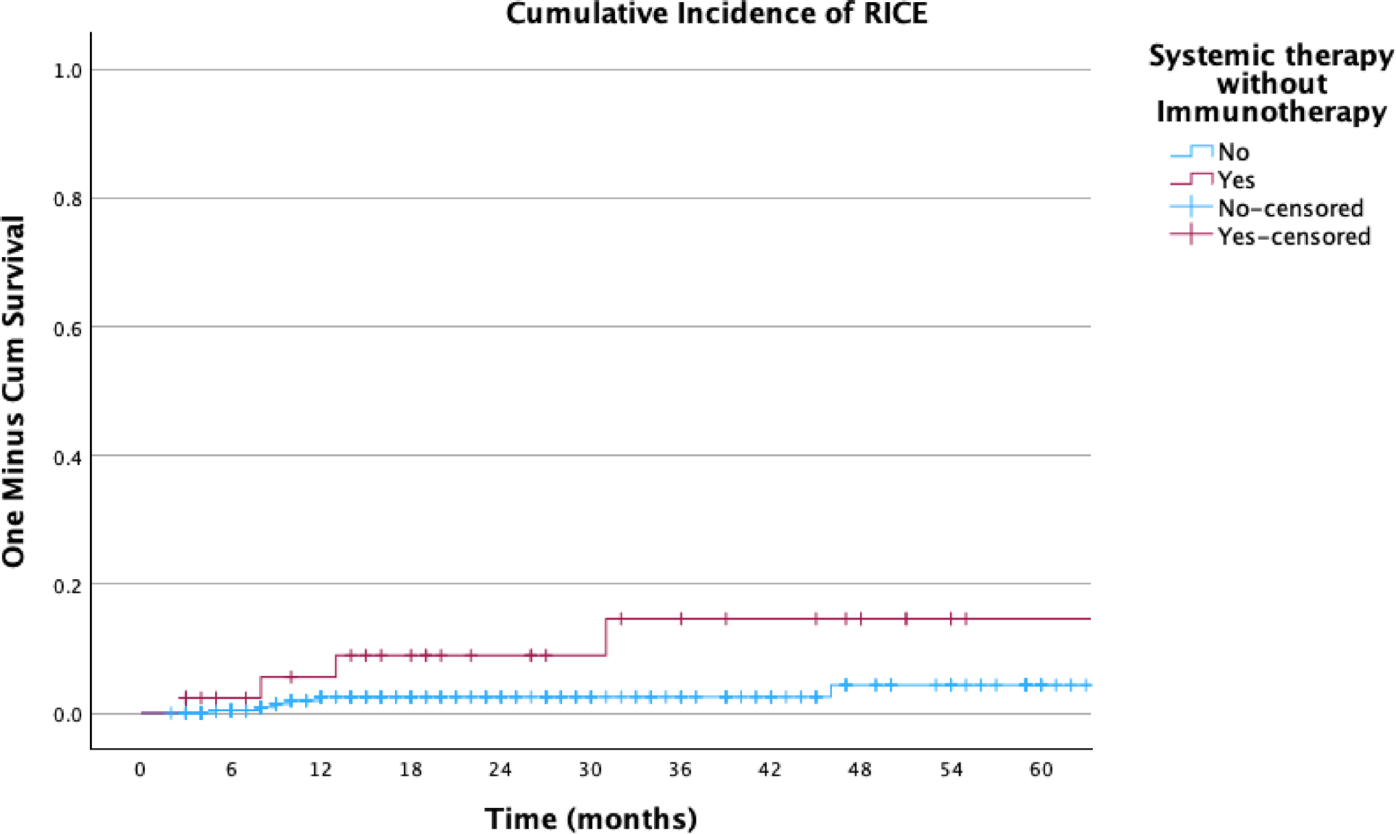
Actuarial incidence of RICE stratified by systemic therapy without immunotherapy

Figures 3, 4 and 5 illustrate the actuarial incidence of RICE: Fig. 3 presents the time to RICE according to lesion location, Fig. 4 shows the overall cumulative probability of RICE, and Fig. 5 presents the incidence stratified by the use of systemic therapy without IT.

**Fig. 6:**
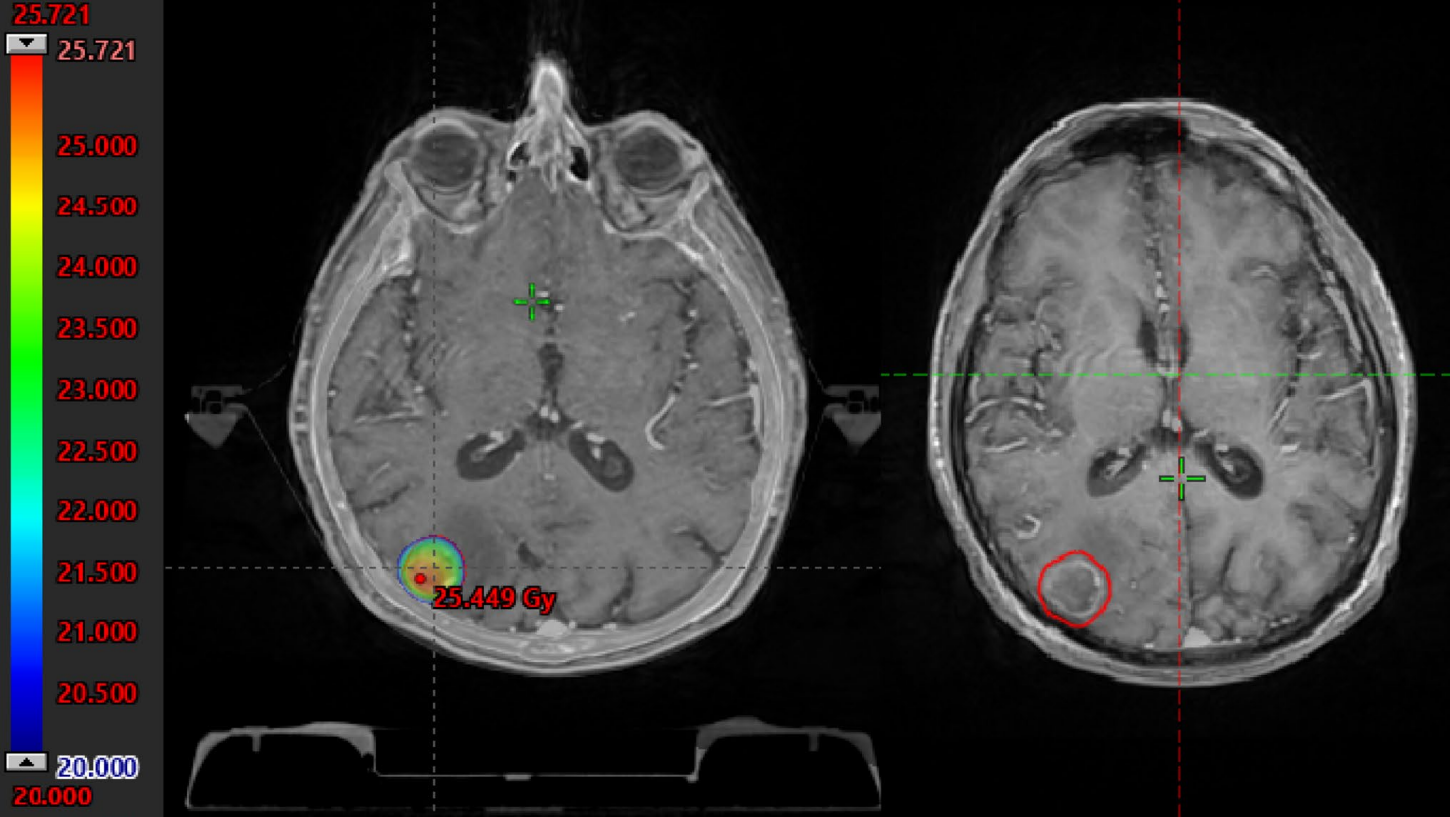
Composite axial T1-weighted contrast-enhanced MRI images. The left panel shows the pre-radiotherapy (RT) scan with no signs of contrast enhancement or edema, and the right panel shows the post-RT scan demonstrating new-onset contrast enhancement and perilesional edema within the high-dose region, consistent with RICE. The red contour delineates the 80% isodose line from the original treatment plan

**Fig. 7:**
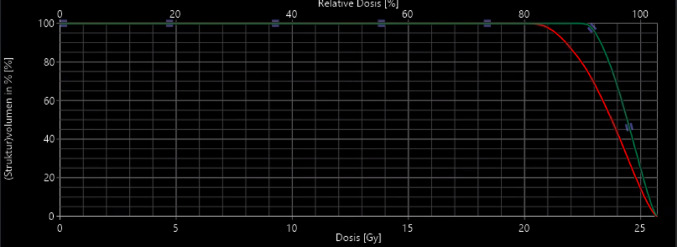
Dose-volume histogram (DVH) of the target volume associated with the lesion shown, illustrating the dose distribution and coverage

Several other variables, including prior irradiation (HR = 0.699, *p* = 0.734), RT under IT (HR = 0.733, *p* = 0.631), BED (HR = 1.237, *p* = 0.096), and RT modality (SRS vs. FSRT; HR = 0.163, *p* = 0.163), did not reach statistical significance. However, trends observed for factors such as BED and the number of RT courses (HR = 3.408, *p* = 0.076) may warrant further investigation in larger cohorts (Figs. 6 and 7).

Of note, conventional predictors like planning target volume (PTV), age at treatment, number of metastases, tumor location or the type of primary tumor did not demonstrate a significant correlation with RICE incidence in this cohort.

These results highlight the potentially deleterious role of systemic therapy administered without IT and suggest that IT may offer a protective effect against radiation-induced cerebral injury. Future prospective studies are necessary to validate these findings and to explore underlying biological mechanisms.

## Discussion

RICE is a well-documented complication of SRS and FSRT, with reported incidences ranging from 3 to 24% in the literature [4, 5]. In our study, RICE was observed in 10 lesions (3%), consistent with lower-end estimates from previous studies. This relatively low incidence may reflect advances in treatment planning and delivery, including the use of high-precision imaging and customized stereotactic masks to minimize radiation exposure to healthy tissue [10]. Additionally, the use of FSRT in a significant proportion of our cohort (37.6%) may have contributed to the lower RICE rate, as fractionation allows for the repair of sublethal radiation damage in normal tissue [12].

Our univariate Cox regression analysis identified systemic therapy without IT as a significant predictor of RICE (HR = 4.161, *p* = 0.027). This finding aligns with previous studies suggesting that systemic therapies, particularly those without immunomodulatory effects, may exacerbate radiation-induced tissue damage [13]. Previous studies have associated chemotherapy agents such as temozolomide and targeted therapies like bevacizumab with an increased risk of RICE, possibly due to their effects on vascular integrity and tissue repair mechanisms [14].

In contrast, IT, which enhances the immune system's ability to repair damaged tissue, may have a protective effect against RICE [15]. However, further research is needed to elucidate the mechanisms underlying this association and to determine whether specific systemic therapies are more likely to contribute to RICE.

Interestingly, our study found no significant correlation between RICE and OS. In our cohort, the mean OS was 31.4 months, and the median OS was 21.5 months (range: 3–209 months), as shown in Table 4. This suggests that while RICE can cause significant morbidity, it does not necessarily impact long-term survival outcomes.This finding is consistent with prior studies reporting that RICE, while debilitating, is often manageable with corticosteroids, hyperbaric oxygen therapy, or surgical intervention [16]. However, the impact of RICE on quality of life and neurological function remains a critical consideration in patient care. Patients with RICE may experience chronic symptoms such as cognitive decline, motor deficits, and seizures, which can significantly affect their daily functioning and overall well-being [17]. Therefore, even though RICE may not reduce survival, it remains an important complication to prevent and manage.

The lack of significant predictors such as radiation dose, target volume, or prior irradiation in our analysis contrasts with some previous studies, which have identified these factors as key contributors to RICE risk [12, 18]. This discrepancy may be due to differences in patient populations, treatment protocols, or FU durations. For example, our cohort included a relatively high proportion of patients receiving FSRT, which may have mitigated the risk of RICE compared to single-fraction SRS [15]. Additionally, the use of advanced imaging techniques, such as perfusion-weighted MRI, may have improved our ability to distinguish RICE from tumor progression, reducing the apparent incidence of RICE in our study [10].

While conventionally FSRT is delivered in 3–5 fractions, our institutional protocol included extended fractionation regimens (up to 10 fractions) in selected cases. This approach was clinically justified based on lesion size, proximity to eloquent or critical brain structures, or concerns about peritumoral edema. Such adaptations reflect real-world treatment decisions made in a multidisciplinary setting. Given this inherent heterogeneity within FSRT protocols, the comparison of outcomes between SRS and FSRT in our cohort is presented in a descriptive manner and should be interpreted cautiously. No direct equivalence between fractionation schedules is implied.

Our study has several limitations, including its retrospective design and relatively small sample size, particularly for RICE events. The retrospective nature of the study limits our ability to control for confounding variables, and the small number of RICE cases may have reduced the statistical power to detect significant predictors. Additionally, the use of different systemic therapies and the lack of detailed dosimetric data may have influenced the results. Future prospective studies with larger cohorts and longer FU periods are needed to validate our findings and further explore the predictors of RICE.

Despite these limitations, our study provides valuable insights into the incidence and predictors of RICE in patients treated with SRS or FSRT for BMs. The identification of systemic therapy without IT as a significant predictor of RICE highlights the need for careful patient selection and treatment planning, particularly in patients receiving systemic therapies. Advances in treatment planning, imaging, and systemic therapies offer promising avenues for reducing the risk of RICE and improving outcomes for patients with BMs.

Future studies should consider incorporating validated patient-reported outcome measures (PROMs) and neurocognitive assessment tools to better quantify the functional and quality-of-life impacts of RICE.

## Conclusion

In patients with BMs treated with SRS and FSRT, RICE was observed in 3% of the cohort, confirming it as a rare but clinically relevant complication. Systemic therapy without IT emerged as a significant predictor of RICE, emphasizing the importance of careful patient selection and treatment planning, particularly in those receiving systemic therapies.

Although RICE can cause neurological symptoms and affect quality of life, our analysis showed no significant correlation between its occurrence and OS. This suggests that RICE, while a potential side effect of treatment, does not appear to influence long-term survival outcomes.

Advancements in treatment planning, imaging, and systemic therapy offer promising strategies to mitigate the risk of RICE. Future studies should aim to identify additional risk factors, clarify the underlying biological mechanisms, and develop effective approaches for the prevention and management of RICE in patients with BMs.

## Data Availability

No datasets were generated or analysed during the current study.

## Abbreviations

RICE: Radiation-induced cerebral contrast enhancement
SRS: Stereotactic radiosurgery
FSRT: Fractionated stereotactic radiotherapy
RN: Radiation necrosis
SRT: Stereotactic radiotherapy
WBRT: Whole-brain radiotherapy
LC: Local control
PFS: Progression-free survival
OS: Overall survival
FU: Follow up
GTV: Gross tumor volume
CTV: Clinical target volume
PTV: Planning target volume
Gy: Gray
SF: Single fraction
BED: Biologically effective dose
RT: Radiotherapy
MRI: Magnetic resonance imaging
CT: Computed tomography
CTCAE: Common terminology criteria for adverse events
FLAIR: Fluid-attenuated inversion recovery
IFP: In-field progression
OFP: Out-field progression
BMs: Brain metastases
NCP: Neurocranial progression
BSC: Best supportive care
